# Intimate partner violence as a factor in contraceptive discontinuation among sexually active married women in Nigeria

**DOI:** 10.1186/s12905-020-00990-y

**Published:** 2020-06-17

**Authors:** Joseph Ayodeji KUPOLUYI

**Affiliations:** Department of Demography and Social Statistics, Obafemi Awolowo University, Ile-Ife, Nigeria

**Keywords:** Contraceptive, Discontinuation, Intimate partner violence, Nigeria

## Abstract

**Background:**

In spite of the well-established associations between socioeconomic and demographic factors and the high rate of contraceptive discontinuation among sexually active married contraceptive users, little is known in Nigeria about the relationship between contraceptive discontinuation and sexually active married women who have experienced Intimate Partner Violence (IPV).

**Methods:**

The 2013 Nigeria Demographic and Health Survey data on women’s reproductive calendars and domestic violence were used to investigate the relationship between IPV and contraceptive discontinuation in a year period. A weighted sample size of 1341 women in a union in the domestic violence module, who have experienced IPV, and are using any contraceptive and are not sterilized in the 12 months periods was analyzed using frequency tables and chart, Pearson’s chi-square test, and binary logistic regression model.

**Results:**

The results showed that women who have experienced any type of IPV are 1.28 times more likely to have discontinued contraceptive use although they are still at risk of becoming pregnant (aOR = 1.28, CI: 1.15–1.91; *p* < 0.05) than those who have not experienced IPV. The tertiary level of education (aOR = 3.94, CI = 1.67–9.29; *p* < 0.05), unemployed status (aOR = 1.97, CI = 1.07–3.62; *p* < 0.05), and higher marital duration of 20 years and above (aOR = 4.89, CI = 2.26–10.57; *p* < 0.05) significantly influenced women who have experienced any types of IPV to discontinue contraceptives even though they are still at risk of becoming pregnant than those who have not experienced IPV.

**Conclusion:**

The study revealed that women who have experienced any form of IPV were significantly influenced by their education, occupation, the number of living children, and marital duration to discontinue contraception while still at risk of becoming pregnant. Thus, the study concludes that intervention programmes aimed at increasing contraceptive prevalence rate should be mindful of IPV which may affect women’s use of contraceptives.

## Background

Contraceptives are devices planned to prevent sexually transmitted infections (STIs), high-risk, mistimed, and unintended pregnancy [1]. Its use is fundamental in reducing maternal and child mortality [1, 2]. The use of contraceptives could prevent as big as 40% of maternal deaths [3]. A key component in addressing sustainable development goals and the stalled total fertility rate in many developing countries is to increase access to contraceptive services [4, 5]. Unfortunately, contraceptive prevalence rate (CPR) is persistently low in many developing countries particularly in Nigeria [4]. An increase by as much as two percentage points per year in CPR for modern contraceptive methods has been canvassed via family planning in Nigeria [6]. Nevertheless, the percentage of currently married women (15–49) who are currently using a method of contraception is very low in the country [6]. The overall prevalence rate of contraceptives among Nigerian women is 15%; showing an increase of only two percentage points between 2003 and 2013 [5]. This increase is far below the expected set target of raising CPR to 36% by the year 2018 in Nigeria [6].

There is a high unmet need for contraception in Nigeria [5]. Literature has shown a great proportion of Nigerian women who want to avoid pregnancies and/or postpone births but are not using contraceptives [5, 6]. Many contraceptive users have discontinued despite being at risk of having unintended pregnancy [7, 8]. The contraceptive stoppage, while women are still at the risk of unintended pregnancy, has negative reproductive health consequences [9, 10]. In developing countries, for instance, half of all unintended pregnancies are terminated illegally in unsafe conditions with resulting morbidities or mortality [11]. In addition, children born from unintended and unwanted pregnancies have tendencies of facing developmental, psychosocial, and growth challenges [12]. Thus, high rates of contraceptive discontinuation implies that family planning has little effect on total fertility rates reduction and could only bring about a high unmet need for contraception [13]. Reasons for contraceptive discontinuation as well as the different brands of contraceptives discontinued among married women are well documented and have shown that contraceptive discontinuation often occurred within a year of adoption of a method [14, 15] while the median duration of the use of contraception before discontinuation gets closer to 2 years [7]. Side effects, method failure, menstrual disruption, husband/spousal disapproval, menopause, fear of infertility, and desire for more children are cited as reasons for contraceptive discontinuation [7, 16–18]. Other contributing predictors include age, marital status, parity, education, place of residence, occupation, household income, number of under-five children, and men’s occupation [15, 19, 20]. In spite of the well-established associations between socioeconomic and demographic factors and contraceptive discontinuation [6, 7, 9, 10, 14, 15], little is known about the relationship between contraceptive discontinuation and women who have experienced Intimate Partner Violence (IPV) in Nigeria. IPV refers to some behaviour within a relationship that causes physical, emotional/psychological, or sexual harm to those in the relationship [21]. A substantial and growing body of literature has shown that the levels of involvement of partners, as well as their opposition to family planning methods, are crucial factors in using, switching, and discontinuation of contraceptives [22–24]. Research on reproductive coercion suggests the influence of some forms of IPV on women’s ability to continue using contraceptives [9]. The attitude of men in a relationship may stimulate the use, switching, and discontinuation of contraceptives [25]. Abused women may be somewhat submissive to men in reproductive health decision-making and therefore use or not use contraception [26]. However, high rates of contraceptive discontinuations may mar previous efforts by the government in increasing family planning and contraceptive prevalence rate among married women in Nigeria [6].

Studies have highlighted that the CPR for any family planning methods has been stagnant at a very low rate of 16% in Nigeria since 1993 [6]. One of the contributing factors for this persistently low CPR is contraceptive discontinuation [10]. Studies on the predictors of contraceptive discontinuation among women have revealed that fertility desires, parity, education, socio-economic status, and age are central to discontinuation in contraceptive use [25–27]. For instance, women’s age significantly influenced their decision to have more children [27]. Young unmarried contraceptive users are more likely to discontinue using contraceptives once they married and intend to have children than older married contraceptive users who need to space or limit the number of children [28]. Also, women’s educational level is significantly related to women’s risk of contraceptive discontinuation [28]. Women who are rural dwellers are more likely to discontinue contraception than urban women [27]. Religious affiliations also influenced the use and discontinuation of contraceptive [29, 30]. Literatures on contraceptive discontinuation have established that reversible contraceptive methods are linked to high rates of discontinuation [7, 31, 32]. Prior studies on the relationship between contraceptive discontinuation and IPV showed mixed results [7, 31, 32]. Discontinuation rates, however, differ reliably by methods [7, 17, 18]. For instance, a limited sign of association was found between IPV and the odds of contraceptive discontinuation [9]. Other studies found inconsistent direction and/or vary associations by a form of IPV [9, 10]. A negative association was observed between contraceptive use and IPV [9, 33–36]. A majority of women who has experienced IPV have more difficulty in using contraceptives to regulate their fertility aspirations [10]. However, this depends on the type of violence. For instance, a negative and a positive association in Tajikistan and Jordan respectively were found between sexual violence and the odds of contraceptive discontinuation [9]. Likewise, a positive association was reported between physical violence and contraceptive discontinuation in Egypt [9]. Empirical Literature has shown that a substantial number of women who have experienced IPV have a higher likelihood of discontinuing using contraception [37, 38]. On the other hand, IPV may have motivated some women who experienced it to use contraceptive method secretly [27, 30, 33], and/or seek for voluntary sterilization [9, 14–16]. Nevertheless, other studies reported no association between experienced IPV and contraceptive discontinuation [29, 33, 39, 40]. However, women who have ever in their lifetime experienced IPV are more likely to have ever used modern contraception sometime in the past than those who have not reported IPV [39, 40]. Nonetheless, the duration of experiencing IPV and the degree of IPV may affect a woman’s contraceptive choices [34, 41, 42]. Thus, it is against this background that this study aimed at examining IPV as a factor affecting contraceptive discontinuation in Nigeria.

## Methods

### Study area

Nigeria is made up of the six geo-political zones, the thirty-six states, and the Federal Capital Territory (FCT), Abuja. There are 774 constitutionally recognized local government areas (LGAs) and about 374 identifiable ethnic groups, with the Hausa, Yoruba, and Igbo as the main ethnic groups. Each LGA is sub-divided into localities. The 2013 Nigeria Demographic and Health Survey (NDHS) is the sixth survey of its kind to be executed by the National Population Commission (NPC) [Nigeria]. The survey provides up-to-date information on background characteristics of the respondents and health indicators at the national level as well as for urban and rural areas.

### Study design

The study used the 2013 Nigeria Demographic and Health Survey (NDHS) women’s individual recode (IR) file. The NDHS is a nationally representative, population-based cross-sectional survey.

### Sampling technique

The survey employed a three-stage stratified and multistage cluster area sampling techniques. Stratification was done by separating each state into urban and rural areas. The survey covered 40,680 households from 904 Primary Sampling Units (PSUs) in both rural and urban households. The PSU was considered as a cluster in the survey based on the enumeration areas (EAs) from the 2006 census EA frames. In the first stage of selection, 893 localities were selected with probability proportional to size. The second stage involved the selection of one EA from the clusters with an equal probability selection. In a few localities, more than one EA was selected. This resulted in the selection of 372 EAs from the urban areas and 532 from the rural areas. In the third stage, a total of 45 households were selected through equal probability systematic sampling from each rural and urban cluster. In all, 40,680 households were sampled for the survey; 23,940 in the rural areas and 16,740 in the urban areas. All women age 15–49 who were either permanent residents of the households in the sample or visitors present in the households on the night prior to the survey were eligible and interviewed [5].

Ethical procedures and questionnaires for the 2013 DHS were approved by ICF Institutional Review Board (IRB) in the United States and the National Ethics Committee in the Federal Ministry of Health of Nigeria. ICF IRB guarantees that the survey conforms with the U.S. Department of Health and Human Services regulations for the protection of human subjects (45 CFR 46), whereas the host Nigeria IRB ensures that the survey complies with laws and norms of the country. Both written and signed informed consent was obtained from all the participants before participation in the survey, and information was collected anonymously and confidentially (NPC [Nigeria] and ICF International. 2014).

### Data collection

The study extracted data on women who experienced IPV in the past 1 year along with data from the reproductive calendar on contraceptive use. Firstly, the contraceptive calendar collects information on reproductive and contraceptive use histories. The study used a contraceptive calendar because it has been acknowledged as the most improved source of data to study contraceptive use dynamics [7, 43–45]. The calendar records the history of contraceptives used month by month in the last five calendar years prior to the survey plus the survey’s year. To avoid bias that may be introduced by unnoticed pregnancy, the last 2 months to the survey and the month of the interview were left out in the analysis [32]. The episodes of contraceptives used (a period of uninterrupted use of contraceptives (in months) that may or may not have ended) from 3 to 59 months prior to the date of the survey was used as the unit of analysis in this study. The DHS data from the reproductive calendar are recorded in a series of string variables (vcal variables) for each of the columns in the calendar [46]. Thus, the study converted these string variables into event data files to make each reproductive event (duration in months) becomes one observation in the dataset. The event variables were used to calculate the discontinuation of a contraceptive method. The study used string functions commands in Stata 14 to transform and restructure the calendar data into a single month and then created event data files for analysis.

Secondly, the DHS violence module collects information on all forms of domestic violence. Women ages 15–49 years were asked about their experience of violence. Thus, data on women who have experienced IPV were selected and interviewed in the domestic violence module were included in this study. Derivation of the analytical sample was done by excluding 11,118 women who have never married nor formerly married; 5656 women who were not selected, women selected but not interviewed, and those selected but privacy not permitted in the domestic violence module; and lastly, 18,762 women who/whose husbands were sterilized. Therefore, out of the total sample of 38,948 women aged 15–49 years in the IR data file, a weighted sample size of 1341 currently married women or cohabiting women with a male partner in the domestic violence module who have experienced IPV, and were using any contraception 12 months prior to interview and were not sterilized or declared infecund were analyzed.

### Outcome variable

The outcome variable, contraceptive discontinuation refers to the disruption of the use of contraceptives for at least 12 months before the survey. It was operationalized as a dichotomous variable, coded ‘1’ for women who are using contraception 12 months before the survey, but stopped using it before the end of the 12-month period and coded ‘0’ otherwise. This classification of discontinuation was further disaggregated based on whether discontinuation occurred even though they are still at risk of unwanted pregnancy or not. Discontinuation while still at risk was coded “1” if women want to become pregnant, discontinued because of health concerns/side effects, stopped because of method inconvenience, wanted a more effective method, cost, lack of access, or stopped using contraceptives as a result of husband opposition and ‘0’ otherwise. On the other hand, discontinuation while not at risk of pregnancy coded as “1” if women discontinue because they want to become pregnant or for infrequent sex/husband away, marital dissolution/separation, difficult to get pregnant) and ‘0’ otherwise.

### Explanatory variables

The principal explanatory variable, Intimate Partner Violence (IPV) was measured in the DHS using three levels namely: physical violence, sexual violence, and emotional violence (see d106–8 of the domestic module of the 2013 NDHS). Women were asked three emotional violence, seven physical violence, and three sexual violence questions on their partner or husband’s actions that indicate IPV in the last 12 months preceding the survey using the revised Conflict Tactics Scale (CTS) [47]. On emotional violence, they were asked to state whether their husbands humiliate, hurt or harm, or insult them. On physical violence, they were asked to state whether husbands push/shake/throw something at them, slap, twist arm or pull hair, punch with his fist or with something that could hurt, kick/drag/beat them, try to choke or burn, threaten or attack with a knife, gun or any other weapon. Finally, they were also asked questions on sexual violence to find out if their husband forced them to have sex, perform any other sexual acts, and threats in any other way to perform sexual acts against their will. A ‘yes’ response indicates that the act took place and a ‘no’ indicates the act did not take place. Responses to all the types of violence are factored into one single binary explanatory variable as experienced IPV ‘1’ and never experienced IPV ‘0’. Other explanatory variables included in the model were woman’s age, age at first birth, education, employment, wealth quintile, region, place of residence, religion, living children, marital status, husband education, husband employment, desire for more children, husband desire for children, and marital duration.

### Statistical analyses

The data was analyzed by employing both descriptive and inferential statistics using the Stata statistical package version 14 [48]. Frequency distributions were used to describe the background characteristics of the respondents. Pearson’s chi-square test was employed in the bivariate analysis to examine the association between contraceptive discontinuation and experience of IPV at *p* < 0.05 level of significance. The domestic violence module sample weights and the Stata complex survey (svy) commands were used to cater for stratified sample design and the effect of oversampling or undersampling of some regions or areas as recommended by DHS [5]. In the multivariable analysis, a binary logistic regression model was employed to examine whether there is any statistically significant association in the odds of contraceptive discontinuation and IPV while controlling for the socio-demographic characteristics of the respondents and other correlates of discontinuation. The selection of variables included in the model was guided by theory and literature. Tests for collinearity among variables were performed using the variance inflation factor < 0.5. All predictor variables that were significantly correlated with the contraceptive discontinuation were retained in the logistic model. Also, physical violence, sexual violence, and emotional violence were combined and factored into one binary explanatory variable: IPV. The factors loaded strongly. Thus, the study, analyzed only the first episode of contraceptive use in the observation period and in the period of discontinuation rather than the timing of discontinuation. Finally, a logistic model was used rather than a hazard model to avoid the error of underestimating the true relationship between contraceptive discontinuation and IPV when censoring is very serious [10].

### Measurement of variables (see Table 1)

**Table 1:** Measurement of variables

Intimate Partner Violence (IPV)	Generated from yes/no questions suggesting emotional, physical, and sexual violence. A composite factor analysis was performed on the three forms of violence and coded as experienced, and not experienced.
Contraceptive discontinuation	Defined as the interruption of contraceptive use for one month or longer by women who had used a contraceptive method in the past 12 months but discontinued at least once without switching to another method. It was coded as not discontinued, and discontinued.
Discontinuation while still at risk	It was defined as discontinuing for reasons other than wanting to become pregnant or no longer at risk of becoming pregnant (e.g. health concerns/side effects, method inconvenience, wanted a more effective method, cost, lack of access, or husband opposition). It was coded as yes, and no.
Discontinuation while not at risk	It was defined as women who discontinue because they want to become pregnant or for other fertility-related reasons (e.g. infrequent sex/husband away, marital dissolution/separation, difficult to get pregnant). It was coded as yes, and no.
Marital duration	Marital duration is calculated by subtracted age at the time of marriage from age at the time of the survey, in completed years. This measure in whole years was further reduced to 5-year categories as a standard variable in the standard recode datasets. The categories are 0–4 years, 5–9 years, 10–14 years, 15–19 years, 20–24 years, 25–29 years, and 30 years or more.
Marital status	Marital status was classified as: never married, married, living with a partner, divorced, widowed, and separated in the DHS. It was recoded as married or living with a partner, never married, and formerly married
Education	Women’s highest level of education attained. It was grouped as none, primary, secondary, and tertiary
Employment	Employment status of women 12 months prior to the survey. Recoded as currently working, and currently not working.
Number of living children	A number of living children at the time of the survey. It was recoded as 0, 1–4, and 5+
**Husband characteristics**	
Husband age	This was measured in whole years in the dataset. It was recoded as 20–29 years, 30–39 years, 40–49 years, 50–59 years, and 60 years or more.

## Results

### Descriptive statistics

#### Contraceptive discontinuation

The result in Table 2 shows the proportion of respondents who have discontinued contraceptives use during the 12-month observation period. Out of 1341 respondents (analytic sample), who discontinued the use of contraceptive method within the 12-month observation period, only 20.11% discontinued contraceptive method while at risk of pregnancy and 79.89% discontinued contraceptive method while not at risk of pregnancy.

**Table 2:** Percentage distributions of respondents by their contraceptive discontinuations and selected characteristics during the 12-month reference period

Characteristics	Contraceptive Discontinuation not at risk N (%)	Contraceptive Discontinuation at risk N (%)	Total N (%)
**Total**	1071 (79.89)	270 (20.11)	1341 (100.0%)
**IPV**			
Never Experienced IPV	815 (76.09)	193 (71.70)	1008 (75.21)
Experienced IPV	256 (23.91)	77 (28.30)	333 (24.79)
**Education**			
No Education	60 (5.62)	7 (2.33)	66 (4.95)
Primary	237 (22.11)	79 (29.25)	316 (23.55)
Secondary	549 (51.23)	130 (48.35)	679 (50.65)
Tertiary	225 (21.04)	54 (20.08)	280 (20.85)
Employment*			
Working	207 (19.46)	23 (8.68)	230 (17.28)
Not working	856 (80.54)	246 (91.32)	1102 (82.72)
**Husband’s age**			
20–29	87 (8.10)	16 (5.94)	103 (7.67)
30–39	543 (50.73)	81 (30.11)	624 (46.58)
40–49	342 (31.92)	125 (46.53)	467 (34.86)
50–59	75 (6.98)	33 (12.15)	108 (8.02)
60 and above	24 (2.27)	14 (5.27)	38 (2.87)
**Number of living children**			
None	1 (0.09)	6 (2.12)	7 (0.50)
1–4	870 (81.20)	186 (68.90)	1055 (78.73)
5 and above	200 (18.71)	78 (28.98)	279 (20.77)
**Marital duration**			
0–4	210 (19.59)	25 (9.42)	235 (17.55)
5–9	377 (35.17)	61 (22.43)	437 (32.61)
10–14	270 (25.20)	73 (27.13)	343 (25.59)
15–19	142 (13.25)	62 (23.09)	204 (15.23)
20 and above	73 (6.78)	48 (17.94)	121 (9.03)

*Missing values excluded

#### Reasons for contraceptive discontinuation

**Fig. 1:**
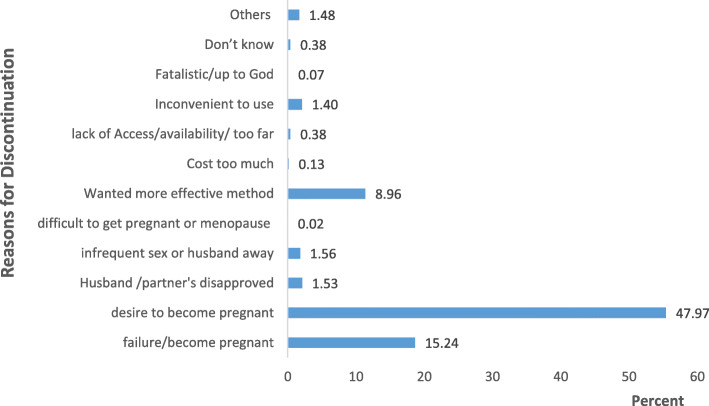
Reasons for the last contraceptive discontinuations during the 12-month reference period

On the reasons for the last discontinuation during the 12-month reference period, the result in Fig. 1 shows that contraceptive discontinuations often occur as a result of the desire to become pregnant (47.97%), method failure (15.24%), need for a more effective method (8.96%), infrequent sex (1.56%), partner’s disapproved (1.53%), and inconvenient to use (1.40%) among others.

#### Intimate partner violence (IPV)

Table 2 also shows the proportion of the sampled populations who have discontinued contraceptives use during the 12-month observation period. The result shows that a quarter (24.79%) of the respondents have experienced IPV. Among those who have discontinued contraceptives use while still at risk of becoming pregnant, 28.30% have experienced IPV while 71.70% have not experienced IPV.

#### Socio-demographic characteristics

Table 2 presents the percentage distribution of respondents’ selected socio-demographic characteristics. As shown in the table, more than half (50.7%) of the study population had at least secondary education, about 83% were working, and nearly 47% had husband’s aged 30–39. The majority of the respondents (79%) had between 1 and 4 living children. Half of the respondents (50%) had less than 10 year’s marital duration. Also, the results revealed that about 68% of the respondents who have discontinued contraceptive use, had at least secondary level of education, 91% were currently working, and 69% had 1–4 living children. Also, about 47% of respondents whose husbands’ aged 40–49, and 27% whose marital duration is between 10 and 14 years, have discontinued contraceptives use while still at the risk of becoming pregnant.

#### Bivariate and multivariable analyses

The unadjusted odds ratio in Table 3 shows the associations between contraceptive discontinuation while at the risk of becoming pregnant and the selected covariates. The results show that, there is no statistically significant relationship between any form of IPV experience and contraceptive discontinuation while still at risk of becoming pregnant (OR = 1.26, CI = 0.88–1.79; *p* > 0.05). The results on the relationship between selected explanatory variables and contraceptive discontinuation show a statistically significant relationship between contraceptive discontinuation while still at risk of becoming pregnant and level of education, employment status, husband’s age, number of living children and marital duration. Women’s level of education was statistically significant with contraceptive discontinuation while at risk of becoming pregnant. For instance, while comparing with women with no education, the odds of contraceptive discontinuation while at risk of becoming pregnant decreases with level of education. It was also highest among women with primary level of education (OR = 3.19, CI = 1.44–7.09; *p* < 0.05). Furthermore, women who are currently not working have higher odds of 2.54 times than women who are currently working [RC]. The odds of contraceptive discontinuation while still at risk of becoming pregnant increase with an increasing husband’s age. Wives whose husbands are aged sixty and above (60+) are 3.17 times more likely to discontinue the use of contraceptives while still at risk of becoming pregnant than others (*p* < 0.05). No statistically significant associations are noticed among women aged below 40 years. On the number of living children, a significant association was found among women with more than five living children and those with one to four (1–4) living children (OR = 0.04, CI = 0.01–0.07; *p* < 0.05 and OR = 0.07, CI = 0.02–0.09; *p* < 0.05 respectively). A statistically significant relationship was found between marital duration and contraceptive discontinuation while still at risk of becoming pregnant. The odds of contraceptive discontinuation while still at risk of becoming pregnant are higher among women who have been married longer compared to women who have been married less than 5 years (0–4 years).

**Table 3:** Unadjusted and adjusted odds ratios with 95% confidence interval (CI) from the logistic regression model predicting contraceptive discontinuation while still at risk in the 12 months prior to the survey

Characteristics	Unadjusted Model		Adjusted Model 2	
	OR	95% CI	aOR	95% CI
**IPV**				
Not experienced IPV	1.00 (RC)		1.00 (RC)	
Experienced IPV	1.26	0.88–1.79	1.28 *	1.15–1.91
**Socio-Demographic Characteristics**				
**Education**				
No Education	1.00 (RC)		1.00 (RC)	
Primary	3.19**	1.44–7.09	3.73**	1.61–8.65
Secondary	2.28*	1.03–5.02	3.37**	1.48–7.67
Tertiary	2.30*	1.02–5.18	3.94**	1.67–9.29
**Employment**				
Working	1.00 (RC)		1.00 (RC)	
Not working	2.54***	1.48–4.36	1.97*	1.07–3.62
**Husband’s age**				
20–29	1.00 (RC)		1.00 (RC)	
30–39	0.81	0.42–1.56	0.65	0.32–1.32
40–49	1.99	0.96–4.12	1.12	0.48–2.60
50–59	2.38*	1.04–5.45	0.97	0.37–2.55
60 and above	3.17*	1.15–8.76	1.33	0.38–4.67
**Women Fertility History and Preference**				
**Number of living children**				
None	1.00 (RC)		1.00 (RC)	
1–4	0.04**	0.01–0.07	0.02***	0.00–0.14
5 and above	0.07 *	0.02–0.09	0.01***	0.00–0.12
**Marital Duration**				
0–4	1.00 (RC)		1.00 (RC)	
5–9	1.33	0.73–2.43	1.37	0.71–2.62
10–14	2.24**	1.21–4.15	2.06*	1.01–4.19
15–19	3.63***	2.00–6.56	3.23**	1.53–6.80
20 and above	5.50***	2.93–10.33	4.89***	2.26–10.57

* p < 0.05 ** p < 0.01 *** p < 0.001 RC Reference Category, *OR* Odds Ratio, *CI* Confidence Interval, *aOR* Adjusted Odds Ratio

Table 3 presents the adjusted odds ratios for contraceptive discontinuation while still at risk of becoming pregnant and the selected covariates. The odds of women who experienced any form of IPV are statistically significantly associated with contraceptive discontinuation while still at risk of becoming pregnant. Women who have experienced any form of IPV are 1.28 times more likely to have discontinued contraception while still at risk of becoming pregnant (aOR = 1.28, CI = 1.15–1.91; *p* < 0.05) than those who have not experienced IPV. The unadjusted odds ratio shows no significant association between any form of IPV and discontinuation. Also, women who are currently not working have 97% greater odds of contraceptive discontinuation while still at risk of becoming pregnant than women who are currently working (aOR = 1.97, CI = 1.07–3.62; *p* < 0.05). Compared to the unadjusted model, there was a strong positive significant association even though somewhat decreased in magnitude. Also, women with a higher number of living children have lower odds of contraceptive discontinuation while still at risk of becoming pregnant than women with lower or no living children. For instance, women with more than five (5+) living children have 99% lower odds of discontinuing contraceptive use (aOR = 0.01, CI = 0.00–0.12; *p* < 0.05) than women with no living child. Similarly, women with one to four (1–4) living children have 98% lower odds (aOR = 0.02, CI = 0.00–0.14; *p* < 0.05) than women with no living child. In contrast, the level of significance is of small magnitude and feeble. Women with an increasing marital duration has a significantly higher odd of contraceptive discontinuation while still at risk of becoming pregnant. The odds of contraceptive discontinuation while still at risk of becoming pregnant are higher among women who have been married longer compared to women who have been married less than 5 years (0–4 years). The result shows that women who have been married for more than 20 years are 4.89 times (aOR = 4.89, CI = 2.26–10.57; *p* < 0.05) likely to have discontinued contraception while still at risk of becoming pregnant than women who have been married less than 5 years (0–4 years). In contrast, the significant associations of contraceptive discontinuation while still at risk of becoming pregnant with marital duration in the bivariate (unadjusted models) continue and are stronger in the multivariable model.

## Discussion

The study examined factors affecting contraceptive discontinuation. Precisely, the study focused on whether women who have experienced any form of IPV are more likely to discontinue the use of contraceptives while at risk of becoming pregnant. The study revealed that within the 12-month observation period, one-fifth (20.11%) of the analytical sample has discontinued contraceptive methods while at risk of becoming pregnant. Most discontinuations however occurred because of a desire to become pregnant (47.97%), method failure (15.24%), and the need for a more effective method (8.96%) among others and is similar to the previous studies [7, 17, 18]. The adjusted odds ratio of discontinued contraception while still at risk of becoming pregnant from a binary logistic regression model revealed that women who have experienced any form of IPV are 1.28 times more likely to have discontinued contraception while still at risk of becoming pregnant than those who have not experienced IPV. This finding is consistent with previous findings [37, 38, 45] which revealed that women who have experienced IPV were more likely to experience discontinuation in their contraceptive use. Lack of sexual autonomy, vulnerability to contraceptive failure, and fear of side effects might be responsible for the relationship. After controlling for socio-demographic and fertility history and preference covariates, women who are not working have 97% greater odds of contraceptive discontinuation while still at risk of becoming pregnant than women who are working. This is consistent with findings in earlier studies [10, 15] that women’s occupation is significantly related to the discontinuation of contraceptive while still at risk of becoming pregnant. Women discontinue contraception use for fertility and those who are unemployed are more likely to have more children than those employed. As expected, women with a higher number of living children have lower odds of contraceptive discontinuation while still at risk of becoming pregnant than women with lower or no living children. In addition to this, women with more than five living children have marginally 99% lower odds than women with no living child. Similarly, women with one to four (1–4) living children have 98% lower odds than women with no living child. This could be a result of the previous method’s failure, fear of side effects and husband’s opposition which might have influenced women with a higher number of living children to discontinuation contraception. The fact that these were significant predictors complements several previous studies [10, 15] and it identifies the importance of some number of living children as a predictor of contraceptive discontinuation. Though, the relationship was weak and marginal. As expected, women with an increasing marital duration has significantly higher odds of contraceptive discontinuation while still at risk of becoming pregnant. The odds of contraceptive discontinuation while still at risk of becoming pregnant are higher among women who have been married longer compared to women who have been married less than 4 years (0–4 years). For instance, women who have been married for more than 20 years are 4.89 times more likely to discontinue contraception while still at risk of becoming pregnant than women who have been married less than 4 years (0–4 years). This contradicts a study which found the lowest odds of contraceptive discontinuation while at risk of becoming pregnant among women who have been married longer compared to couples who have been married less than 5 years [10]. One possible explanation though difficult to explain, however, could be due to inertia, side-effect, and fear of complication among other reasons. Women with fewer number of children are likely to be younger with academic/career pursuit therefore not likely to discontinue using contraceptives.

### Study strengths and weaknesses

This study used the domestic violence module and reproductive calendar. The possibility of underreporting of violence by respondents should be taken into consideration while interpreting the findings using the domestic violence module. Also, the reproductive calendar was used to measure contraceptive behaviour for the last 5 years. But, in this study, contraceptive discontinuation is limited to 12 months because the domestic violence module covered only 12 months. Another limitation is that the results of this study should be interpreted with caution because DHS is a cross-sectional data and thus, causality cannot be established. Finally, the data were collected retrospectively, and thus there is the possibility of recall bias and other biases. Despite these limitations, the survey is nationally-representative and population-based. Thus, it allows the generalization of the findings to the whole population.

## Conclusion

The study concludes that women who have experienced any form of IPV were significantly influenced by their education, occupation, the number of living children, and marital duration to discontinue contraceptive use while still at risk of becoming pregnant than those who have not experienced IPV. Thus, intervention programmes aimed at increasing contraceptive prevalence rate should be mindful of various forms of IPV which may affect women’s use of contraceptives.

## Data Availability

The DHS individual recode (IR) data set was used for this study and is available from the DHS Program archive at www.measuredhs.com. Permission to use the data was obtained.

## Abbreviations

IPV: Intimate Partner Violence
CPR: Contraceptive Prevalence Rate
DHS: Demographic and Health Survey,
NDHS: Nigeria Demographic and Health Survey
PSU: Primary sampling unit
NPC: National Population Commission
LGA: Local Government Area
EA: Enumeration Areas
FCT: Federal Capital Territory
IR: Individual Recode
CTS: Conflict Tactics Scale
aOR: Adjusted Odds Ratio
OR: Odds Ratio
CI: Confidence Interval
RC: Reference Category
IRB: Institutional Review Board
